# Next-generation sequencing in Brazilian MODY patients: a pilot study

**DOI:** 10.1186/1758-5996-7-S1-A257

**Published:** 2015-11-11

**Authors:** Lílian Araújo Caetano, Lucas Santos de Santana, Antônio Marcondes Lerário, Márcia Nery, Alexander Augusto de Lima Jorge, Milena Gurgel Teles

**Affiliations:** 1 USP, São Paulo, Brazil

## Background

Maturity-Onset Diabetes of the Young (MODY) is the most common form of monogenic diabetes. Genetic analysis is required to confirm the diagnosis. Conventional genetic testing uses Sanger sequencing. Currently, Next-generation sequencing (NGS) has proven to be cost-effective. To date, there is no NGS study for MODY in Brazil.

## Objective

To validate a new assay for molecular diagnosis of MODY using targeted-NGS.

## Materials and methods

We have completed a pilot project including 7 unrelated subjects with MODY phenotype. Genetic sequencing was performed using Illumina NGS platform (MiSeq), allowing analysis of 13 MODY genes simultaneously. All exonic and intronic regions of these genes were evaluated. Two cases were not tested before. In other 5 cases, a previous analysis using Sanger sequencing of GCK and HNF1A genes had been done. Three subjects had already a genetic diagnosis of MODY by Sanger method and were selected to validate NGS Results. And the other 2 cases had typical clinical features but negative Sanger analysis.

## Results

**Figure 1:**
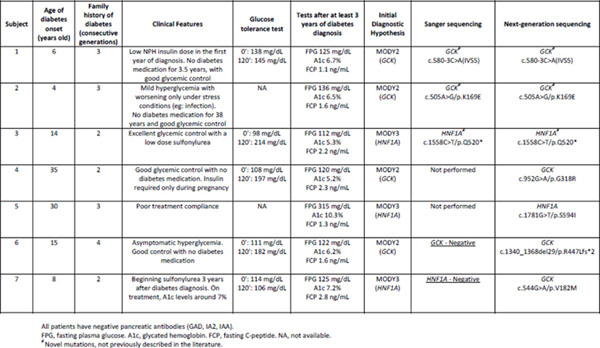
Mutations identified by NGS in Brazilian subjects with MODY phenotype.

In all 7 cases analyzed, NGS was able to detect the mutations related to MODY, and in those 2 previous negative Sanger sequencing, it has allowed us to confirm the diagnosis of this type of diabetes (Figure 1). Considering these 2 negative Sanger cases, one had a mild hyperglycemia detected at 15 yo, non-progressive during 4 yrs. of follow-up, normal C-peptide, negative beta cell antibodies, and also a family history of similar phenotype. Previous Sanger testing for GCK had yielded a false negative result, because the mutation was a large deletion located at the end of the last exon of GCK, which impaired the analysis by Sanger, however was detected by NGS. The second negative Sanger subject had diabetes since 8 yo, low BMI, negative antibodies, detectable fasting C-peptide, and a mother with asymptomatic hyperglycemia. This patient had been using sulfonylurea with good glycemic control. Sanger sequencing for HNF1A was negative. NGS identified a mutation in GCK, already described in the literature as pathogenic.

## Conclusions

In our pilot project, targeted-NGS was able to confirm MODY diagnosis in all cases submitted to Sanger sequencing (3 positive controls and 2 previously negative cases). In other two not tested before, this new method could identify the pathogenic variants. Thus, NGS can be considered an effective tool for diagnosing clinical suspicious cases of MODY, appearing to be a promising technique.

